# The Hospital Spirit in National Affairs

**Published:** 1916-12-16

**Authors:** 


### THE HOSPITAL SPIRIT IN NATIONAL AFFAIRS.

THERE is probably no congregation in the country which is not already aware that Sunday, December 31, is the day fixed for the annual collections in the churches on behalf of the Red Cross. It is not on behalf of that fund that these articles are written. The whole nation and every newspaper is full of enthusiasm for the cause, and realises that the need of the Red Cross is not a stationary, but a growing one. Our business here is to remind the public that the requirements of the voluntary hospitals are equally constant, and that, indeed, they have a double claim from the fact that they are providing not only for the wounded men, but for their wives and children and relatives. It is gratifying to realise that public opinion, represented by the municipalities, realises this and desires to express its gratitude to the voluntary hospitals for the double burden that they are supporting to-day. Thus the City Council of Coventry has just voted £1,000 to the Coventry and Warwickshire Hospital for the part it has played in treating the sick among civilians and soldiers alike. If a municipality can be so moved to action as to loose its purse-strings at a time like this, private persons will hardly be backward in generosity.

We hear at this moment of the crisis which has arisen in the financial affairs of a London children's hospital; and since Christmas is above all things the children's festival, the old appeal of the Christmas season will hardly fail to stir those alike who love children for their own sake and those who realise that the child to-day is doubly worth our care, since it is on his or her young shoulders that the burden of reconstruction will fall when once peace is renewed and good will between nation and nation becomes once more a fact of everyday life. The victories of peace, of which Milton wrote, are victories over disease, the forces of Nature, lazy thinking, and all the difficulties and temptations which beset man in his fight against the lower forces outside and inside himself. These are the permanent things, the constant tests of courage, while militarism and war are only the evil interludes when man himself shuts the gates of mercy on mankind. There is no story in the world which moves the human imagination more than St. Luke's story of the shepherds in the fields by night and of the birth which took place in a stable because there was no room for mother and Child in the inn. Every mother and child who does not receive hospital treatment in time of sickness is represented by the mother and Son who were turned away on the first Christmas night. Every person to-day who is unmoved by the appeal of those who stand in need of this treatment is turning away the Madonna and her Son as surely as those who could find no room for them in Bethlehem. This is felt more deeply in hospitals than outside, and the fact is worth mentioning because it explains why so much trouble is taken over hospital decorations and festivities at Christmas, and why the happiest place on Christmas Day should always be the hospital ward. Let no reader take this assertion on trust. Let him go to the nearest up-to-date hospital and see for himself how Christmas should be kept in hospital.

Such a visit will show the inquirer that the atmosphere of a hospital ward is no mere creation of the holiday spirit. Its gaiety and gentleness, its consideration and personal care, speak of the permanent basis of the work, the spirit of which is busy day in, day out throughout the seasons, only to burst out in the glory of flowers and fairy-lamps on the great day of Christmas itself.

The gospel of personal service, which the urgency of war has made a living reality to thousands who never awoke to its meaning before, is one which has always been the message of the voluntary hospitals. These institutions are largely managed by those who proudly give as offerings their time and skill; and they are staffed by those who, though paid, are taught from first to last to regard their calling as a true vocation, and not merely as a professional career. It is, indeed, because they are a living protest against the motive of commercialism that they rely upon voluntary gifts to pay their way. The public subscribe money, the men of business give their time, the doctors seek nothing but experience, and the nurses are taught that character is of even larger importance than professional skill in attaining to the standard set by Florence Nightingale. The public, then, must realise that in helping to maintain our voluntary hospitals they become part of this spiritualising force, and that everything for which a voluntary hospital stands is the opponent of the temper which we are now trying to exorcise from European affairs. The voluntary hospitals remain a heritage from our fathers. Florence Nightingale embodied their spirit. Dickens placed his eloquence at their service. The poorest and the richest alike rely on them for help. The gospel that they began by preaching has been forced upon the nation as the only gospel which can win this war. Who can fail to respond to the call they make this Christmas for large and generous gifts to carry on for another year achievements so great?

